# AI ethics through a decolonial lens: what does AI ethics look like if we take seriously the push to decolonise it?

**DOI:** 10.1007/s00146-026-02935-9

**Published:** 2026-02-23

**Authors:** Selena Nemorin, Beatrice Bonami

**Affiliations:** 1 School of Education, Culture & Society, Monash University, Melbourne, Australia; 2 School of Social and Political Sciences, University of Edinburgh, Edinburgh, United Kingdom

**Keywords:** Artificial Intelligence, Decolonial AI, Ethics, Global South

## Abstract

This paper offers an exploratory examination of the emerging discourse and practices on decolonising AI ethics. Whilst the field of AI ethics has made substantial progress in proposing normative frameworks for responsible innovation, these frameworks remain predominantly grounded in Western epistemological traditions, foundations which have limited the capacity of AI ethics to engage with plural ontologies and non-Western moral traditions, particularly those emerging from the Global South. Drawing from decolonial and critical data studies, this paper interrogates what an alternative, decolonial orientation to AI ethics might entail in both conceptual and practical terms. We contend that mainstream AI ethics frameworks fail to recognise epistemic diversity and to establish dialogical relations with non-Western knowledge systems, thereby reproducing colonial hierarchies of thought and governance within technological design and deployment. An answer to the question of decolonising AI ethics is not simple or straightforward and will, therefore, not be single handedly delivered in this paper, whilst we acknowledge the situatedness and limitations of our study. Whilst we understand that there are many other realms to examine for a decolonial AI ethics (such as critical infrastructure, labour, and political economy), we decide to focus on the following reflections: (1) AI as a means to redress power asymmetries and structural exploitation; (2) AI grounded in local data sovereignty and equitable epistemic participation; and (3) AI conceived as relationally entangled with humans, more-than-humans, and the Earth. These reflections foreground the material and ecological dimensions of data—its extractive infrastructures, environmental externalities, and geo-political inequalities—as central to the ethical assessment of AI. Through a literature-based exploration of these domains, we argue that decolonising AI ethics requires moving beyond the universalist and procedural tendencies of Western ethical paradigms towards pluriversal, situated, and ecologically embedded forms of ethical reasoning. The paper concludes by outlining how these reflections can serve as a theoretical scaffold for rearticulating AI ethics in and from the Global South, resisting the uncritical replication of Northern frameworks and opening pathways for epistemic plurality in global AI governance.

## Introduction

Given the challenges facing our planet, Artificial Intelligence (AI) is an evolving field with the capacity to tackle some of humanity’s most pressing concerns. Through modelling large datasets, identifying patterns, and automating tasks, AI is promising a number of ways in which we can address issues we once thought challenging to resolve, including breakthroughs in fields like medicine and material sciences; personalisation of education systems; information verification and validation; developing more sustainable practices; and optimising energy consumption (Chui et al. 2018). However, despite these benefits, AI systems also possess the potential to do harm, such as the risk of privacy violations and biases against historically marginalised groups (Borgesius 2018; Noble 2018).

These challenges have caught the attention of an array of individuals and groups, including those from government, non-government, and industry. As a result, ethical frameworks have been developed, highlighting the aims of AI being used to nurture human flourishing, thereby creating new opportunities for the development of a sustainable society. As Floridi et al. (2018) write, AI may enable self-realisation, the ability for people to flourish in terms of their own characteristics, interests, potential abilities or skills, aspirations, and life projects, if developed thoughtfully, improving and multiplying the possibilities for human agency. AI algorithms and codes under the scrutiny of newly launched regulations have attempted to mitigate the harms potentially inflicted onto historically marginalised groups. For instance, biased algorithms might engender discrimination and other kinds of incorrect decision-making that may result in systematic error and unequal access to AI (Luxton and Watson 2023; Eubanks 2018) and ethical frameworks for trustworthy AI have attempted to account for these challenges through commonly cited principles, such as fairness, privacy, explainability, safety, and accountability.

However, mainly conservative and mainstream conceptions of these principles are developed with alternative perspectives mostly neglected. This form of ethics scrutinises design decisions and often ignores cultural and political situatedness (Haraway 1988) of such systems. In fact, ethics itself is a Western construct, being a dominant narrative (Adams 2021) that appears to be supported by corporations (and international organisations that also represent corporate interests) to comply with human rights standards. The EU AI Act (European Commission 2024) is an example of this, adopting a risk-based framework that presumably creates a more permissive innovation environment for European companies to “catch up” in the global AI race. Whilst current methodologies of fairness have not suitably accounted for the socially constructed nature of race, for example. Rather, these principles have ingrained in their conceptualisations of race as a fixed attribute. This has consequences inasmuch as when race is approached as an attribute instead of a structural, institutional, and relational phenomenon, it minimises the structural components of algorithmic unfairness (Hanna et al 2020).

Moreover, as Hagendorff (2022) claims, most of the key AI ethics initiatives hold methodological challenges; they use a principled approach and determine a set of rules and standards that are claimed to have an effect of governance on AI research and development. Yet, studies on metaethics have shown that numerous approaches, largely principled and grounded on the deontological view, have many shortcomings (Hagendorff 2022). There are limited reinforcement mechanisms, often deployed for marketing, and insensitive to diverse contexts, as they do not really have significant influence on the behaviour of practitioners. Mechanisms as such often fail to consider the technical complexity of AI and the value chains embedded in its networks of production, since they tend to be technologically deterministic, using concepts that are often too abstract to be put into practice. In addition, guiding para-government frameworks have been grounded on a Eurocentric view of ethics (Nemorin 2024).

Scholars have also identified the geo-political effects of the AI Empire (Hao 2025) which “functions as a set of technologies, a mode of production, a web of social relations and material resources, a culture, a knowledge base, and a worldview” (Tacheva and Ramasubramanian 2023, p. 4). In this capacity, AI is not merely positioned as a system or lifecycle but is seen as a complex ecology which serves to enact geo-political dominance for nations such as the United States (U.S.) and China. For instance, AI’s origins are deeply rooted in U.S. military, economic, and political ambitions, primarily aimed at reinforcing U.S. empire, as explicitly outlined in America’s AI Action Plan where “The U.S. is in a race to achieve global dominance in artificial intelligence” (U.S. Government 2025, p. 4).

Whilst China presents a formidable competitor in the AI race, the U.S. holds a significant advantage in terms of coercive power and global influence. The U.S. market Global model for data capitalism (Zuboff 2019) is strategically designed to maintain its dominant position, leveraging its resources and established networks to ensure that AI developments align with its geo-political interests. This strategic approach allows the U.S. to dictate the trajectory of AI innovation, using it as a tool to expand its hegemony. As a result, the competition between the U.S. and Chinese AI models is not just about technological superiority but also about the broader geo-political implications of AI, with the U.S. currently holding the upper hand in shaping the future landscape of AI development and application (Couldry and Mejias 2019).

This is apparent in international governance forums, where AI ethics is mainly addressed to the extent that it does not conflict with U.S. interests and directives. Regrettably, given the current conflicts and the emphasis on AI and its origins in militarisation (Haraway and Wolfe 2016), which is consistently omitted from AI ethics discussions, the international community has demonstrated no willingness to halt the inhuman use of AI in warfare, thereby becoming complicit in AI ethics washing (van Maanem 2022). If the design and deployment of AI is to facilitate flourishing, particularly for marginalised global populations as well as the environment (including more-than-human actants), AI ethics needs to be decolonised. The AI decolonisation movement works towards this by highlighting how the AI lifecycle embodies the principles of modernity/coloniality where inequalities are grounded in the process of colonisation (Adams 2021; Mhlambi and Tiribelli 2023), when they could instead be advocating for a different view that is pluriversal (Escobar 2017).

The decolonisation of AI lifecycles is a critical task that aims at addressing the historical legacies of colonialism integrated into contemporary technological production and deployment. This process attempts to challenge dominant Western-centric knowledge systems and bring to the fore diverse knowledge structures, thereby promoting equity in technological fields. Concomitantly, we ought to observe that AI ethics decolonisation has to tackle the geopolitics of how digital market models have asserted their historical and imperial dominance worldwide. It is, therefore, crucial to acknowledge that the geo-political order of AI must be challenged to allow other nations a voice in shaping the future of humanity. And as Ricaurte (2022) observes, AI ethical frameworks should be examined as strategic and discursive tools that advance the interests of nations and corporations developing AI, which are embedded within their colonial structures.

In this regard, we seek to pull apart hierarchies and exclusions, whilst acknowledging diverse ways of knowing and methodologies, endeavouring to make technological knowledge more responsive to the needs of groups that have been impacted by the effects of colonialism. At the same time, we look at environmental and more-than-human aspects regarding AI’s networks of production (Crawford 2021) arguing that without an ethical framework that encompasses the complexity of the AI ecosystem, decoloniality might remain an abstract principle. Envisioning a scenario in which the AI ecosystem does not compete with human and more-than-human existence, thus fostering a sustainable approach to its embedded materiality (Krenak 2020; Crawford 2021). With this in mind, the present paper attempts to consider the regularly ignored dimensions of AI ethics, although still leaving out fields of critique, such as critical infrastructure, labour, and political economy, since they deserve a separate space to be properly addressed. We move the discourse around a decolonial AI ethics forwards in two sections. First, we examine the literature on decolonial perspectives on AI. Second, we explore what a decolonised AI ethics might look like if we take the process of decolonisation seriously (acknowledging the limitations of our approach and opening room for future investigation). As a result, we expect that this paper serves as a grounding endeavour to rethink not only the course of AI policy, but also the routes of ethics studies that can be qualitatively responsive to the Global South.

## A decolonial perspective

Decolonial theory is grounded in the debates about modernity/coloniality as espoused originally by scholars in Latin America (Mignolo 2007, 2011; Escobar 2007; Dussel and Ibarra-Colado 2006; Quijano 2000), becoming prominent in the critique of the Eurocentrism of modernity and claims of universality. Modernity is described as a phenomenon that denotes the primacy of Europe from the instant the Americas were found; coinciding with the prelude for the first industrial revolution in 1760. It refers to the emergence of discourses, practices, and institutions that have developed over the past few hundred years from European ontological and cultural colonisation. Here, the world and knowledge developed on the grounds of an ontology of modernity seemed to become a universal ontology. This universality gained authority over other worldviews and practices.

The notion of coloniality is a direct extension of colonialism, defined by Horvath in 1972 as a form of domination by individuals or groups over the territory and/or behaviours of other individuals or groups. Coloniality thus represents the enduring legacy and structural implications of colonialism, where the power dynamics and systemic oppression established during colonial rule continue to influence and shape societal relations, culture, and power structures in the post-colonial era. Observers have also argued that coloniality describes how colonialism’s mechanisms for social domination shape power and knowledge, maintaining Western views continue shaping power and knowledge to uphold Western perspectives (Tacheva and Ramasubramanian 2023). Consequently, history and its associated knowledges became a product of the West, a tale about the exploration of no-one-lands.

As Mignolo (2007), writes “modernity, capitalism and coloniality are aspects of the same package of control of economy and authority, of gender and sexuality, of knowledge and subjectivity” (p. 162). This mode of coloniality refers to “the pattern of power which has emerged as a result of colonialism” and is an explicit strategy of epistemological control and domination” (Misoczky 2011, p. 347). The coloniality of knowledge refers to how the West’s knowledge system achieved intellectual and cultural imperialism that suppressed, as well as eradicated alternative knowledge systems and social structures. As Ndlovu-Gatsheni (2013) writes, in colonialism, “the commercial non-territorial empire and the cognitive empire are inextricably intertwined” (p. 208). Something achieved through the method of epistemicide, the murdering of knowledge (Mignolo 2007)—as the imperial conquest took over land by attempting to erase the roots and systems of indigenous wisdom and knowledge.

Following the work of Tacheva and Ramasubramanian (2023), we emphasise modernity/coloniality, because it encompasses both the enduring impact of colonialism and its ideology of racial and technological dominance. This perspective recognises that categories, such as colonialism and neocolonialism, are frequently referenced in discussions about AI. We also draw upon the idea of *data colonialism* (Couldry and Mejias 2019) which speaks to the various ways in which social resources are extracted and appropriated through data; this heuristic is essential for understanding the accumulation of wealth and power by companies that often began as social media platforms indiscriminately collecting data and have now moved into the AI ecosystem. However, our focus on modernity/coloniality can help reveal how the AI lifecycle moves beyond data colonialism’s focus on datafication and data extraction to encompass the various damages these systems inflict upon the world and its populations.

To offer one example, examining AI as a lifecycle can reveal the mechanisms involved in the labour-intensive processes of mining and refining rare-earth minerals used in the hardware that powers AI frequently occur in environmentally/climate stressed and conflict zones (Kara 2024). These processes perpetuate an old colonial/imperialist logic and place an unequal burden of climate change on the majority world, manifesting as a form of climate coloniality (Sultana 2022). We also acknowledge the importance of understanding AI as an Empire (Hao 2025). In this capacity, AI can be seen as a “networked entity comprising multiple axes, agendas, and forces” (Tacheva and Ramasubramanian 2023, p. 2). As Hao (2025) puts it, the AI Empire comprises the histories of racism and colonialism which are informed by a largely Western understanding of technology, commanded by corporations that apply domination dynamics across nations and knowledge domains. Examining AI from this perspective allows one to represent the lifecycle of AI algorithms, including the attached material, knowledge, data, logistical, labour, and political, cultural, economic, and ideological infrastructures (Crawford 2021) and how this ecology functions as a colonising entity.

Additionally, we highlight Birhane’s (2020) concept of AI colonialism which critiques how AI and data-driven technologies perpetuate and amplify colonial legacies. On this view, the deployment of these technologies is not a neutral phenomenon but a continuation of historical colonial patterns, reinforcing power structures that benefit developed nations at the expense of formerly colonised contexts. Data extraction is a fundamental component here, where data from the Global South is often exploited by the Global North to train AI models, creating a cycle where the Global South provides raw materials with minimal returns. Cultural erasure occurs as local knowledge and practices are overshadowed by dominant cultural narratives, impacting Indigenous communities. Economic exploitation is rife as the benefits of AI technologies primarily accrue to developed nations, thus widening the digital divide. Birhane stresses the role of epistemic injustice, where the knowledge of marginalised communities is dismissed, and reinforced by the dominance of Western epistemologies in AI development inequalities.

AI ethics is implicated in modernity/coloniality given the primacy of Western AI ethics frameworks (Jobin et al 2019) as a form of knowledge production and dissemination under what it seems to be a Global Northern normative morality. As a universal idea, AI ethics refers to the responsible development and deployment of artificial intelligence technologies. A field of study which derived from science ethics, an area inaugurated after the catastrophes caused by the Manhattan project (and the invention of the atomic bomb) (Himma and Tavani 2008). The importance of ethics is underpinned by rapid progressions in AI systems and the possible disruptions they may bring to social structures. AI has undoubtedly sparked business gains, but along the way government and industry leaders have had to address a range of ethical dilemmas, such as data privacy issues, algorithmic bias, and public safety concerns. A central question related to these actions is: which ethical principles ought to guide the ethical development of AI systems?

To guide the development and adoption of AI systems, governments have coordinated AI ethics frameworks, which more or less address levels of risk an AI system can cause, then inputting an ethical allegiance by the developers to ultimately avoid harming humans (European Commission 2024). However, these frameworks have not necessarily included voices from the Global South. For example, do Silicon Valley companies follow the same principles that might guide developers in diverse cultural contexts? Are these concerns relevant to countries with different cultural, religious, and social norms (Corrêa et al 2023)? Thus far, there have been a number of scholars who have examined ethics guidelines (Corrêa et al 2023; Hagendorff 2022; Zeng et al. 2018; Jobin et al. 2019), and the publishing of AI ethics frameworks (such as the one developed by UNESCO 2021,1) tries to bring a holistic approach to what a fair production and deployment of AI might look like. Some of these papers and materials have offered systematic reviews of the existing documentation around AI ethics, which we will briefly present next. The present paper, however, does not intend to offer a comprehensive investigation of documents on AI data ethics. Rather, it seeks to examine the ways in which scholars have positioned AI ethics and highlights how such an ethical framework might be imagined from a decolonial perspective.

## AI ethics

Data-based discrimination is implicated in how new technologies impact human rights and economic equity (Eubanks 2018). Scholars have espoused critical perspectives specific to AI that have been used to discuss social and ethical implications of these systems. Much of this research explores biases in algorithms. Angwin et al (2016), for example, expose the bias inherent in algorithms that predict rates of recidivism in the US with these systems identifying black populations as more prone to commit a crime than their white counterparts. Similarly, Buolamwini and Gebru (2018) write about how machine learning algorithms can discriminate against classes such as race and gender by presenting an approach to evaluate bias present in automated facial analysis algorithms and datasets in terms of phenotypic subgroups: they find that there is significant disparity in accuracy to classify darker and lighter females and males in gender classification systems.

Furthermore, private interests and the status of a handful of internet search engines result in biased algorithmic systems that privilege whiteness and discriminate against people of colour (Noble 2018). Other scholars have investigated how the use of AI technologies have dispossessed the marginalised (Hanna et al. 2020) and how data are being extracted from individuals by platforms and smart objects, translating human’s everyday lives into data points and selling this information back to us. On this view, this form of datafication is reminiscent of colonialism’s appropriation of bodies and natural resources (Couldry and Mejias 2019).

The role of ethical principles and their effects on underprivileged groups have also been discussed by scholars in terms of a decolonial perspective. Ali (2016) asks: Does computing need to be decolonised, and if so, how should such decolonisation be effected? He argues that in order for the colonial impulse in computing to be overturned, it is necessary for developers and practitioners to consider their geo-political and body-political orientation when designing, building, researching, or theorising about computing phenomena and embrace the ‘decolonial option’ as an ethics. This means thinking about how to design computer systems with and for populations situated in the periphery with a “view to undermining the asymmetry of local–global power relationships and effecting the ‘decentring’ of Eurocentric/West-centric universals” (Ali 2016, p. 7).

Carnevale (2024) advocates for a decolonial perspective to AI ethics. This approach challenges Eurocentric frameworks and emphasises epistemological justice, by recommending the integration of Indigenous cosmologies and local practices to develop AI systems that transcend Western-centric paradigms, ensuring that AI advancements benefit marginalised communities and promote pluriversalism. Carnevale calls for a transition from solely “empowering” AI to acknowledging and empowering human vulnerability within the AI lifecycle, suggesting that emphasising relational autonomy over individual autonomy is crucial for achieving decolonial AI. In addition, the aim is to transcend a singular, universal epistemology and advance towards an “inclusive epistemological innovation” that embraces a variety of ways of knowing, living, and being from marginalised communities.

In their work on decolonial AI, Mohamed et al. (2020) view AI as comprising the cultural, economic, and political life of society. As such, they argue that AI can be understood as both object and subject—as systems of networks and institutions. In this capacity, they raise pertinent questions about values and norms of AI such as: What values and norms should we aim to uphold when performing research or deployment of systems based on artificial intelligence? In what ways do failures to account for asymmetrical power dynamics undermine our ability to mitigate identified harms from AI? How do unacknowledged and unquestioned systems of values and power inhibit our ability to assess harms and failures in the future?

In another work, Mhlambi and Tiribelli (2023) argue that a good number of ethical frameworks have centred on the principle of autonomy as essential for mitigating the potential harms AI might inflict on society, harms which frequently impact the most marginalised groups disproportionately. The principle of autonomy, as it is currently framed in AI ethics, appears to be flawed as it reflects a predominantly liberal notion of autonomy as rational self-determination, rooted in Western philosophical traditions. This form of ethics fails to address the ways in which human autonomy can be violated, it also does not consider the “AI-empowered harms” tied to the presumable project of neocolonisation which affects the most vulnerable groups in a global capacity.

The ability of AI to mediate and automate social relationships through data models, machine learning algorithms, and digital platforms goes against traditional notions of autonomy in which the ability for persons to reason between personal costs and benefits of different actions is weakened. This is because the algorithms and platforms make use of patterns and parameters of data to predetermine and direct a user’s access to material and ability to interact socially. Such weakening of human autonomy undermines human rights as the right to opinion, information, and privacy. That said, even when one adheres to the traditional framing of autonomy through technical and policy implementations, it remains insufficient, because it is unable to gain equitable outcomes for individuals and populations who are negatively affected by the implementation of AI. As Mhlambi and Tiribelli (2023) reason, to confront the harms amplified by AI and maximise its benefits, the principle of autonomy must be reimagined and decolonised from its enlightenment period design.

Whilst the concept of negative externalities originally emerges from the field of economics, when taken from a decolonial stance, it is aligned with the consequences of colonial systems of power, economics, and culture which can come from AI. Such data models affect peripheries of centres of power in economically developed countries such as the Global North. When thinking about AI, negative externalities seem to be caused by the development and application of AI systems which affect subaltern groups such as when AI systems are beta-tested in vulnerable communities. These negative externalities (negative effects on third parties that generally reside outside Western capitalist centres) are rarely discussed in AI ethics (Mohamed et al 2020). Relatedly, there is an underrepresentation of geographic areas, such as Africa, Central Asia, or South and Central America in the AI ethics documentation (Jobin et al 2019). Overall, key debates in the discourse around AI are framed by Western values, contexts, and concerns. However, a robust system of ethics ought to overcome dominant perspectives and emphasise Global South concerns. AI ethics typically fails to do such things.

Scholars have pointed to the fact that AI systems affect the underprivileged in ways that are entrenched in particular values and norms that reside in the colonial residue of modernity (Perrigo 2023; Miceli and Posada 2022). Here, whilst European domination ended with national independence actions in the nineteenth and twentieth centuries, there remain “sedimented” colonial ways of thinking and doing. Ali (2016) notes, these ways of knowing and being are based on “systems of categorization, classification and taxonimization and the ways these are manifested in practices, artefacts and technologies” (p. 2). As such, computing is a colonial endeavour, as Dourish and Mainwaring (2012) point out where the expansionism of ubiquitous computing (ubicomp) is driven by a “colonial impulse.” Ali (2016) claims that “decolonial computing, as a ‘critical’ project, is about interrogating who is doing computing, where they are doing it, and, thereby, what computing means both epistemologically (i.e., in relation to knowing) and ontologically (i.e., in relation to being)” (p. 5). In the next sections, we seek to answer these questions, specifically through an epistemological understanding of how AI ethics has been colonised and how it might be decolonised to reflect a broader view from the perspectives of a range of cultures regarding power imbalances, natural resources deployment, and data poverty.

## What might a decolonised AI ethics entail?

### AI that is designed to redress power imbalances/exploitation

Decolonial perspectives have explored the implications of the production and application of scientific knowledge around the world. As scholars have highlighted, much of this knowledge emerges from countries in the Global North, such as the U.S., the United Kingdom (U.K.), France, and Germany. For Quijano (2000), whilst colonialism has ended, the post-colonial and neocolonial impulse remains marked by a form of coloniality which has taken the guise of social discrimination, outliving formal colonialism to become embedded in succeeding post-colonial and neocolonial social orders sponsored by capitalism itself. Decoloniality’s purpose, instead, is to create pluriversal knowledges where nations are given equitable voice in the process of ethics creation, by designing a world where many worlds can fit (Escobar 2017). Where coloniality failed in giving legitimacy to Global North knowledges and ways of being over other knowledge systems, pluriversality asks for interrupting the hegemony of these dominant knowledge systems, so that Global North and Global South ways of knowing can co-exist equitably (Mignolo 2011). As Anzi (2021) writes: “decoloniality is not a quest for lost origins, but a call for epistemic pluralism that questions the destitution engendered by modernity” (p. 47).

Given the decolonial turn in the study of data and technology, AI technologies are significant when thinking about the pervasiveness of coloniality. In AI ethics, a decolonial approach requires a change in the conception and implementation of systems of ethics. Thus far, ethical standards have largely been driven by Western-centric approaches, giving primacy to the needs of developed nations whilst neglecting the needs of the Global South. A decolonial perspective would challenge dominant approaches by giving voice to the Global South, putting these voices at the centre of decision-making processes whilst also acknowledging the experiences and challenges that pertain to these geographic areas (Cruz 2021). Such an approach emphasises the worth of Indigenous knowledges and local expertise; it accounts for the fact that AI ethics borne in the Global North may not necessarily work for individuals and populations living in the Global South.

Thus far, capitalist knowledge and power asymmetries have led to the silencing of Indigenous knowledges, thereby perpetuating hegemony and creating knowledge dependence. This all too often results in the Global South being forced to import knowledge systems that are incompatible with local contexts. As Ndaka et al. (2024) write: “These systems mirror a particular built-in model of society, which often amplifies existing systemic power asymmetries (p. 9).” AI systems are not likely to incorporate perspectives that are pertinent to Global South communities into AI technologies or systems of ethics. In addition, the languages used to frame issues becomes a challenge when searching for information. For example, values that are embedded in English are different to certain languages that are inherent in Global South contexts (Bird 2020). That said, even if local languages were used, “the initial conception of the system with Euro or Western-centric values is likely to shape local perspectives, even inadvertently, in the Western ideal. The type and quality of data collected only end up reinforcing knowledge hegemony” (Nadaka et al. 2024, p. 9). As such, dominant values are used to establish the way in which AI ethics and AI-related technologies are designed, developed, and deployed.

Related to this is the idea of AI post/neo/colonialism, a term that overlaps with it is “data colonialism,” as a form of injustice. Here, data are extracted from life forms and are particularly concerning in the Global South where rules and regulations to protect individuals and groups from predatory practices are less robust (Couldry and Mejias 2019). For instance, the GenAI wave of attention and investment in 2022 has now seemed to peak, taking scholars and the industry back to its source problem: data. In this capacity, Big Tech companies are also using datafication for social good, which results in the goal of reconfiguring the social domain itself in a manner that presents these companies as “privileged providers of social solutions and privileged purveyors of social knowledge” (Magalhães and Couldry 2021). Here, a commercially driven production of the social good points to a rebalance of power and governance which privileges corporations with large-scale data power, making states and other commercial and civil society actors reliant on these corporations. The corporatisation of the social good holds grave implications for how social life is “known, understood, and governed.” It is situated in the realm of data colonialism where:

…data-driven solutions are rarely offered as part of a range of solutions to social problems, but as the solution, an expression of a new language for defining and solving social problems that replaces all others. No one is asked if they agree with this act of substitution of social knowledge, or its consequences in terms of data collection and processing. In the process, populations’ freedom to define their social good, their version of social knowledge, is overridden. (p. 355)

In keeping with data colonialism, AI colonialism does not seem to acknowledge a social world where marginalised groups are asked to contribute to the generation of new knowledge. Rather, Big Tech acts in accordance with the datafied lifeworlds that are already created and measured. So how might this form of AI colonialism be countered by the project of decolonisation? By fostering Global South regions to take an active role in the design, development, and implementation of AI ethics, global AI has the potential to become more equitable. In addition, the tackling of biases in AI technologies is central to the decolonial project. As has been widely discussed, AI technologies often perpetuate biases grounded in race, gender, or class, for example. Although we understand that eliminating bias is simply not an endeavour that is possible, given that human data by definition is biased, and so would all data collection processes be. That is why mitigating bias, by articulating and acknowledging it, is crucial, so that harm is minimised, or at least, addressed.

In this capacity, AI technologies are designed and developed by a generally homogeneous group largely comprised of young, white, heteronormative, and affluent males, thereby replicating their worldview which is often left unchallenged (Costanza-Chock 2020), augmenting biases and discrimination. These AI biases can circumvent the fairness of AI technologies. When taking a decolonial approach, the onus is placed on the mitigation of these biases to make sure that these technologies do not perpetuate or worsen existing inequalities. Yet, the problems faced by AI are also social and cannot be completely solved by correcting these biases. These problems also emerge, because the constructed worlds have the capacity to change the beliefs of individuals and populations (Birhane 2023). As such, AI systems need to be decolonised epistemically given their ability to reify “Western knowledge and values as universal and the viral reproduction of the colonialist superstructure’s cognitive content” (Mollema 2024, p. 582).

A critical approach to AI ethics which comprises decolonial theories emerges as a resource for examining the complex impact of AI on society, yet not to eliminate its harm. Such a theoretical framework offers a way into evaluating the principles associated with AI technologies and determining whether or not these principles would be beneficial for Global South populations and not prioritising only the Global North. Naturally, an answered question emerges if Global Southern geographies and individuals, specifically experts and technical communities of AI, believe that a decolonial approach to technology might bring a quantitative and qualitative change of how these systems are created and deployed (something to be investigated in further research and literature). Nevertheless, at its core, this approach questions dominant assumptions and ideologies that shape AI ethics, with the capacity to uncover the historical context of AI’s evolution and identifying the relationship between AI, ethics, and the colonial legacy.

### AI that is grounded on local data sovereignty and equity

Data access and governance are central to equity, justice, and the safeguarding human rights. World populations should have the innate right to data sovereignty where they are afforded the capacity to own and make decisions about their data. Whilst there is an inconsistent approach to how academics and policymakers apply the term “data sovereignty”, many of the variations refer to individuals and populations having a control over their data. Issues of access and governance are prevalent when it comes to the ways in which data are used under capitalism which is often in opposition to the rights of the Global South. This tension is particularly felt in the context of Indigenous populations, who, because of their histories of oppression, have been rallying for a different approach to data collection and use—a way data can be collected that is aligned with their values. Data sovereignty aims to “assert some form of collective control on digital content and/or infrastructures” (Couture and Toupin 2019, p. 2308), although we acknowledge the tension in which data sovereignty does not necessarily lead to fair data practices.

Much of the data on Australian Indigenous communities, for example, emerge as a deficit which highlights “disparity, deprivation, disadvantage, dysfunction, and difference” (Walter 2016). The consequences of this process are homogenising and pathologising Indigenous populations to their disadvantage. Data are not value free, and Indigenous perspectives and values have been excluded from data about them collected through dominant epistemological viewpoints and methodologies. Scholars have argued that this kind of approach poses a challenge in that issues faced by Indigenous populations are magnified through derogatory statistical accounts (Snipp et al. 2016). To counter these narratives, the concept of data sovereignty was developed which leveraged international instruments such as the United Nations Declaration on the Rights of Indigenous Peoples (UNDRIP). Existing conceptions of data sovereignty have differing dimensions, but they hold a shared goal of making sure that local stakeholders can assert their self-determination in the context of data stewardship (Couture and Toupin 2019).

Whilst Indigenous populations have long argued for the sovereignty of their lands, the conversation about data sovereignty has been co-opted by governments and multinational corporations who have been focussed on issues of legal jurisdiction (Kukutai and Taylor 2016). What was long missing from these debates is the innate right of Indigenous populations on the collection, ownership, and deployment of data about their communities. As Fraser (2019) writes: “data sovereignty requires that actors in civil society, or in cooperative economic associations, develop principles and practices that explore whether the emergent value of data should be held in common, rather than privatised; destroyed, rather than analysed and brought to market; or stored nearby, rather than exported” (p. 11). Another colonial approach to the topic relies on the field of cyber security, since indigenous stakeholders might lack the necessary infrastructure that comes with protecting data from hacking attacks (something quite often associated with military infrastructure).

Data sovereignty marks a call for communities who want to regain control of their data, pushing against the tide of data colonialism and the harms that this practice inflicts upon historically marginalised populations. The complexity of data sovereignty is attached to issues related to legal ethical aspects of data storage, stewardship, ownership, consent, access, intellectual property rights, and how data are used in the context of research, policy, and practice. Data sovereignty should include dimensions such as:

- Indigenous empowerment to decide who is Indigenous and from whom data should be collected to inform policies;
- Data collection that is aligned with Indigenous values, interests, and priorities;
- Freedom to decide who has access to data collected appropriately; and
- Advocating for respectful data governance, storage, security, and decision-making powers (Moudgalya and Swaminathan 2024, p. 95).

Pragmatically, such dimensions follow a crucial capitalist aspect, in which organisations and governments could then contribute in developing the necessary infrastructure and networks to bring forth these dimensions in a safe manner (as we acknowledge that the question of data sovereignty is usually a notion linked to nation states and data localization). With particular regard to the protection of sensible and sensitive data, an aspect that puts forward a necessary agenda around digital, data, and security literacy. Another angle on these dimensions is about resources and the connection between humans, more-than-humans, and earth, since these advances have environmental costs and bring important criticism towards data materiality (Crawford 2021; Kara 2024). Thus, it is reasonable to assume that even data sovereignty formulations (although crucial to an equitable data society) do not escape resource exploitation, which requires scholars to rethink theories and their applications. Also, it is important here to also add that these environmental costs are most often felt in Global South geographies, putting together two agendas that might have been distant in the past, now intertwined with the rapid space of AI development: data and environmental sovereignty.

### AI that is connected to humans, more-than-humans, and earth

Escobar (2007) writes that the world and all knowledges that have been created on the grounds of an ontology of modernity emerged as a universal ontology. This universal ontology gained traction over life practices, such as the dominance of some humans over other humans, animals, and nature. One of the remnants of coloniality has been the belief that technology is a means of controlling nature. As the pressure of human activities increasingly affects the planet, there is attached to this a hope that AI systems will be able to allow societies to detect and respond to environmental impacts and changes.

Currently, data-intensive projects have promised the capacity to fix our environmental crisis; however, AI technology itself contributes to environmental damage in a number of ways, including the consumption of large amounts of water and electricity, the large-scale extraction of raw minerals, and the creation of e-waste and noise. As such, the inner workings of AI technologies require more resources and resulting in new forms of colonisation, referred to as “techno-molecular colonialism” (Mbembe 2019, p. 32). Techno-molecular colonialism is the combination of colonialism’s tendency to exploit and extract resources from nature with a mode of capitalism that reaches the “molecular depths” of human behaviour and the Earth’s ecosystems. Techno-molecular colonialism holds the power of the “techno-colonial market”, which extends from the neuronal level of behavioural predictions to planetary resource extraction, considering everything in its path as “raw material” waiting to be datafied (Mollema 2024).

From an ecological standpoint, AI must be decolonised because of the environmental distress that AI colonialism places on natural resources. As noted previously, AI has an ecological footprint: it requires vast amounts of resources to power several phases in its networks of production. Mining minerals, such as lithium, tin, cobalt, manganese, and nickel, which are required for the development of AI-related components is becoming a major cause of pollution, affecting the state of flora and fauna (Kara 2024). Vacuuming ground water for the purpose of data centres’ cooling has been leaving natural reserves, rural settlements, and small towns under hydrological stress (Hogan 2015). Overloading electrical grids is causing communities and villages to migrate back to coal energy generation, affecting public health (Brodie 2023). This all leads to a burden placed on Global South nations that are suffering the ecological impacts to fuel the AI progress in the Global North.

The challenge with the current ethical frameworks is that they fail to sufficiently address the complexities of the natural world and how AI systems are impacting on this dynamic in negative ways which enable an extractivist approach to planetary resources, both human and more-than-human. In short, the field of AI ethics does not generally give moral consideration to more-than-human forms of agency (Owe and Baum 2021). Of the key dimensions to AI development, computing and data storage are the more resource intensive, likely posing material risks associated with the distribution of harms. The Global South is potentially left out of economic gains because of significant costs of capital whilst at the same time suffering from the effects of environmental degradation because of these material risks (Crawford 2021).

Whilst there have been studies on AI and sustainability with papers highlighting that sectors like transportation, energy, medical, IT, education, food sector, services, construction, and manufacturing are harnessing the power of AI for sustainable development (Kar et al. 2022), there is space for more critical examination of the negative impact of AI on the environment, the most significant being its energy and fresh clean water consumption and the effects these technological systems have on Global South and Indigenous populations. AI ethics here seem to find a common denominator in which the environmental and developmental costs in the digital industries are being systematically paid by Global South geographies. Something that again follows the colonial heritage, as the Global South, despite holding 85% of the world’s inhabitants, is still seen as an extraction site (both for data and natural resources).

## Discussion

To decolonise AI ethics, then, one must closely examine how colonial ideologies have influenced the design and deployment of AI technologies as well as the ways in which these frameworks have infiltrated the language of AI ethics. In the pursuit of an environmentally centred AI that upholds the dignity of Global South and Indigenous populations, decolonisation also asks for a reimagining of metrics of progress (Diop 1974). Typically, AI ethics have been embedded with principles that leave profit and efficiency intact without looking too deeply into the broader environmental impacts of AI deployment. Decolonising AI ethics, then, would prioritise environmental and social good over the language associated with maximisation of profit. By no means, however, we imply that the pluriversality of knowledge and language is enough, and we acknowledge the persisting need to tackle geopolitics of AI and its data capitalism market models to propose a version of AI ethics that is not too abstract to be put into practice. Yet, we still believe that distancing AI from political economy and neighbouring it, instead, to non-Western epistemologies can be fruitful to include Global South voices in the technology ethics debate: a field that has systematically and historically excluded Southern and non-Western scholarship from digital technology assessment and validation.

A main shift in this approach is to frame AI under its models’ specificity and data and environmental needs instead of how much capital it generates for the private sector, or how much investment it requires from governments to keep it running. Since the GenAI re-launch in 2022, AI has been addressed as a crucial funding pipeline, driving stakeholders from many areas of society to approach it in a timely manner. The pitfall in this verge is the overlooking of data-intensive Large Language Models (LLMs) are (Valdivia 2025). Three years later, after the GenAI wave peaked, we fall back to discuss data availability and quality, arguing that despite LLMs alleged capacity in many knowledge fields, maybe we should be looking instead to more frugal forms of producing and deploying AI (Valdivia 2025).

In the realm of ethics though, some development is noticeable, since regulations and regulatory bodies have now begun to observe the specificities of AI models under the AI umbrella, addressing its requirements in terms of computing capacity and resource deployment. When the AI Act makes a risk classification between models and their potential to human harm or prosperity, it deploys specific language on what these models are and how they can possibly cause harm. Yet, regulations are not yet binding, and the private sector performs a considerable force to remain in charge of their market model: data extraction and stewardship. On a theoretical level, the discourse around AI ethics does seem to have a grasp on these advancements and to be responsive and contributors to the risk classification systems in which many regions are now developing their own, although, again, Western-centred.

Decolonising AI Ethics would involve inviting Global South and non-Western ethics scholars to create a platform in which AI could be discussed under an alternative framework, under its models, natural resources deployment, effects on humans and more-than-humans, data specificity, and with a theoretical base that is responsive to these geographies. Naturally, it is comprehensible that geographies, such as Europe, China, and the U.S., have a robust history and background when it comes to ethics study, whilst Latin America, Africa, and Southeast Asia have been developing the field in the last 100 years. However, we must take the leap in acknowledging this literature and basing AI ethics analysis on Global South and non-Western scholarship, since they might find more ground and responsive aspects to tailoring the analysis to Global South contexts.

Another angle that could improve the decolonisation of AI ethics is regarding AI development, and the fact that many countries—especially in the Global South—struggle to find available and quality data to train models to generate the needed accuracy to operate as AI models. Therefore, a conceptual neighbour of data sovereignty and stewardship is the role that data poverty (Milan and Treré 2020) plays in barring AI development from happening in countries aside from Europe, the U.S., and China. However, there is a deeper argument here tied to how the private sector has instrumentalised the market to create a data gap between AI development and deployment, advocating against federated learning to hold vast amounts of data. Something that even Global North universities point out as a challenge when engaging with models that require tera million data points, such as Large Language Models (LLMs) or Quantum Computing.

Therefore, a decolonial AI ethics could also pay closer attention to the question of open sourcing, prioritising open sourcing of databases and data availability to tackle data poverty. Yet, there is a larger debate that goes in this direction, something initiated with the open source and hacking movements in the 2000s, where Aaron Swartz’ (Reddit founder) condemnation and death sparking how the publishing industry is not compliant with this idea or ideology. The debate persists today, now in the area of data, leaving many spheres empty handed when it comes to finding a sustainable answer that does not solely depend on feeding the private sector. The hacking movements from the 2000s, such as Anonymous, prove that this discussion keeps going back to its source: the power imbalance in which the Global North private sector withholds information capital, therefore, creating a neocolonial ordinance of data capitalism and coloniality (Zuboff 2019). At the same time, the rapid pace in how technology is advancing brings a similar feeling regarding this dependency, with some Global South scholars advocating for a new philosophy of market that is not solely based on profit ideology.

## Conclusion

If AI ethics is to be decolonised, one central aspect to address is how rapidly this industry is evolving and how this swift pace is incompatible with climate and environment. Krenak (2020, 2022) brings to the fore the question that a rupture with technology is not possible, but pacing it and slowing it down is. His point advises how natural resources’ consumption alongside e-waste is not something we can afford as a planet, in an Indigenous framework where body, land, and spirit are indissociable. Of course, this idea goes against AI coloniality and capitalism itself (being a form of neocolonialism), where the message is to produce more and faster than ever before. Despite Krenak speaking about the pacing down of industrial time, it is important to situate that Indigenous knowledges are not homogenous, and their approaches are varied, some even applying an abolitionist approach to AI and progress, such as the Anacés ethnicity in Brazil.

Yet, we keep revolving doors on the same problems, since we are not able to tackle its true core: the capitalist structure of neocolonial industries (Zuboff 2019; Crawford 2021; Hao 2025). Theorists, such as Crawford (2021) and Kara (2024), have predicted that perhaps with the scarcity of fossil fuel or cobalt, the reasoning behind these aspects would reconsider how these industries are designed. But in failing to do that, it is up to us to rethink how this ecosystem can survive without harming humans, a crucial point in AI ethics. But as AI ethics is designed at the moment, the unharmed humans remain location specific, in the Global North, and that is why we need a decolonial approach: since the global majorities are in Global South regions where they keep being harmed by the Global North neocolonial industries (Valdivia 2025). As with any large undertaking, developing a decolonial AI ethics is something possible but effortful (Adams 2021). With the three first steps presented in this paper (adopting a Global South theoretical framework; addressing data poverty on a conceptual level; and approaching a new and slower industrial pace to AI), this task might become a little less overwhelming.

This paper has highlighted the need for a rethinking of mainstream AI ethics, which is inadequate when addressing the complex issues of power imbalances, exploitation, and environmental degradation. By decolonising AI ethics, we can create a more just and fair AI system that prioritises social justice, human rights, and environmental sustainability. This requires a shift in perspective, one that acknowledges the experiences and challenges of Global South communities, values Indigenous knowledges, mitigates bias, and addresses power asymmetries. Such a reimagining of AI ethics would take into consideration three main points as a matter of decolonising normative structures: AI that is designed to redress power imbalances/exploitation; AI that is grounded on local data sovereignty and equity; and AI that is connected and responsive to humans, more-than-humans, and earth.

On this view, decolonial perspectives challenge the dominance of Western-centric approaches to AI ethics, advocating for pluriverse knowledges that give a voice to Global South nations. A decolonial approach challenges the very idea of ethics as a framework for understanding how different cultures approach their relationships with the world and with other beings. At the same time, we recognise the unresolved questions we left untouched and exterior to this paper such as the AI infrastructural dimension (one of the crucial challenges in building digital sovereignty), commercial treaties (which add to digital neocolonialism), and labour (maybe the most pressing concern in the AI networks of production). Decolonising AI ethics, then, requires a shift in perspective and future research and work, examining how colonial ideologies influence AI design and deployment, and prioritising environmental and social justice. This can be achieved by adopting a Global South theoretical framework, addressing data scarcity, and slowing down the pace of industrial development to ensure a more sustainable, frugal and equitable approach to AI.

## Data Availability

No datasets were generated or analysed during the current study.

## Footnotes

1 Link to UNESCO’s AI Observatory and guidelines: https://www.unesco.org/en/artificial-intelligence/recommendation-ethics.
